# A wogonin-rich-fraction of *Scutellaria baicalensis* root extract exerts chondroprotective effects by suppressing IL-1β-induced activation of AP-1 in human OA chondrocytes

**DOI:** 10.1038/srep43789

**Published:** 2017-03-03

**Authors:** Nazir M. Khan, Abdul Haseeb, Mohammad Y. Ansari, Tariq M. Haqqi

**Affiliations:** 1 Department of Anatomy & Neurobiology, Northeast Ohio Medical University, 4209 St Rt 44, Rootstown, OH 44272, USA

## Abstract

Osteoarthritis (OA) is a common joint disorder with varying degrees of inflammation and sustained oxidative stress. The root extract of *Scutellaria baicalensis* (SBE) has been used for the treatment of inflammatory and other diseases. Here, we performed activity-guided HPLC-fractionation of SBE, identified the active ingredient(s) and investigated its chondroprotective potential. We found that the Wogonin containing fraction-4 (F4) was the most potent fraction based on its ability to inhibit ROS production and the suppression of catabolic markers including IL-6, COX-2, iNOS, MMP-3, MMP-9, MMP-13 and ADAMTS-4 in IL-1β-treated OA chondrocytes. OA chondrocytes treated with F4 in the presence of IL-1β showed significantly enhanced expression of anabolic genes ACAN and COL2A1. In an *in vitro* model of cartilage degradation treatment with F4 inhibited s-GAG release from IL-1β-treated human cartilage explants. The inhibitory effect of F4 was not mediated through the inhibition of MAPKs and NF-κB activation but was mediated through the suppression of c-Fos/AP-1 activity at transcriptional and post transcriptional levels in OA chondrocytes. Purified Wogonin mimicked the effects of F4 in IL-1β-stimulated OA chondrocytes. Our data demonstrates that a Wogonin-rich fraction of SBE exert chondroprotective effects through the suppression of c-Fos/AP-1 expression and activity in OA chondrocytes under pathological conditions.

Osteoarthritis (OA), the most common form of joint disorder associated with joint trauma and aging, is characterized by a slow but irreversible, progressive destruction of articular cartilage^1^. The pathophysiology of OA is complex and exhibits not only cartilage degeneration but also changes in the subchondral bone and synovial fluid^2^,^3^. At the molecular level, OA is characterized by loss of metabolic homeostasis due to an imbalance between anabolic and catabolic pathways in chondrocytes^4^. Interleukin-1 beta (IL-1β), a pro-inflammatory cytokine is a potent inducer of many catabolic factors in OA including matrix degrading metalloproteinases (MMPs) and aggrecanases^5^. In addition, IL-1β also induce the expression and production of inflammatory mediators such as IL-6, COX-2, PGE_2_, iNOS and NO that are implicated in OA pathogenesis^6^,^7^,^8^. Further, recent studies have indicated that oxidative stress due to overproduction of reactive oxygen species (ROS) and reactive nitrogen species (RNS) are important components involved in the multifactorial etiology of OA^9^. Currently, nonsteroidal anti-inflammatory drugs (NSAIDs) are commonly used to alleviate the symptoms of OA, however long-term use of such drugs leads to adverse events^10^. Therefore, preparations of medicinal plant extracts rich in flavonoids and polyphenols with a history of usage in the traditional medical practices have been suggested as alternative or complementary therapies because of their minimal side effects and also because they are cheaper, locally available and easily consumable^11^.

*Scutellaria baicalensis*, is one of the most widely used Chinese herbal medicine. Traditionally, its roots have been used for the treatment of inflammation, cancer, allergy, bacterial and viral infections of the respiratory and gastrointestinal tract^12^,^13^. The major bioactive components of root extracts of *S. baicalensis* (SBE) include Baicalin, Scutellarein, Baicalein and Wogonin^14^. SBE has been shown to inhibit lipid peroxidation in rat liver and attenuate oxidative stress in cardiomyocytes^15^. In recent years, natural blend of *S. baicalensis* and *Acacia catechu* were used to alleviate the joint discomfort associated with osteoarthritis of the knee^16^,^17^. Further, recent studies showed that Baicalein and Wogonin, the constituents of SBE suppressed the inflammatory response and inhibited the expression of MMPs in chondrocytes^18^,^19^,^20^. However, the mechanism was not investigated in detail.

Here, we carried out a systematic and comprehensive analysis of SBE to identify the chondroprotective and anti-inflammatory constituents of SBE by activity guided fractionation utilizing preparative HPLC and tandem mass spectrometry techniques. Our results showed that the most active fraction of SBE significantly inhibited the IL-1β induced oxidative stress, inflammatory response and matrix degradation in human OA chondrocytes and suppressed the IL-1β induced release of s-GAG by human cartilage explants. Further, molecular mechanisms attributed to anti-inflammatory and chondroprotective properties of the active fraction was investigated against IL-1β induced activation of mitogen-activated protein kinase (MAPK) and activation of transcription factors NF-κB and activator protein-1 (AP-1) using a well-defined *in vitro* system.

## Results

### SBE Fraction 4 (F4) exert potent chondroprotective effects in human OA chondrocytes

**Figure 1:**
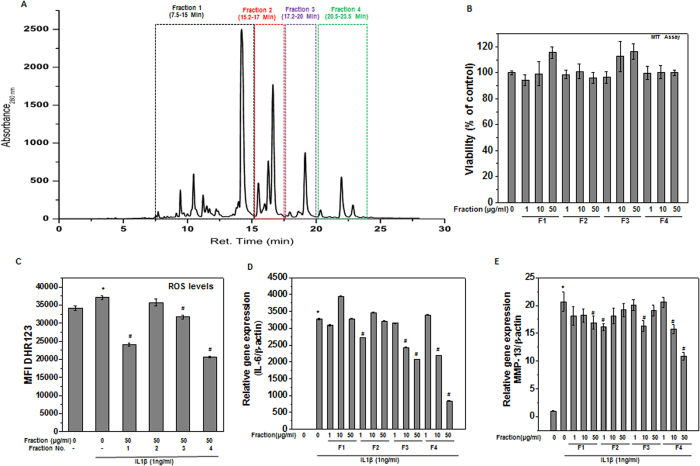
SBE Fraction 4 (F4) showed potent chondroprotective effects in human OA chondrocytes. (**A**) HPLC chromatogram of ethanolic extract of S. baicalensis. Fractions were collected based on indicated retention time. (**B**) SBE fraction (s) was not toxic to human OA chondrocytes. Human OA chondrocytes were treated with indicated concentration (1–50 μg/ml) of SBE fraction (F1 or F2 or F3 or F4) for 24 h and cells viability were measured by MTT assays. Chondrocytes treated with 0.1% DMSO served as control. Viability was expressed relative to control cells. Data points represent mean ± SD from three subjects. (**C**) SBE fraction 4 (F4) inhibited IL-1β induced ROS production in OA chondrocytes. Human OA chondrocytes were treated with SBE fraction (F1 or F2 or F3 or F4) (50 μg/ml) for 2 h, then stained with DHR-123 (5 μM) for 0.5 h at 37 °C, and stimulated with IL-1β (1 ng/ml) for 5 minutes at 37 °C. Fluorescence emission was measured at 535 nm. Bar graph shows relative fluorescence units indicating ROS levels. (**D–E**) SBE fraction 4 (F4) inhibited IL-1β induced mRNA expression of IL-6 and MMP-13 in OA chondrocytes. Primary human OA chondrocytes were pre-treated with SBE fraction (s) for 2 h followed by treatment with IL-1β (1 ng/ml) for 16 h. At the end of treatment chondrocytes were harvested, total RNA were isolated, reverse transcribed to cDNA and mRNA expression of IL-6 (**D**) and MMP-13 (**E**) were measured by quantitative PCR using the TaqMan assay system. Expression of β-actin was used as endogenous expression control. Bar graph represent mean ± SD from three subjects **p ≤ 0.01, as compared to control, # ≤ 0.01, as compared to IL-1β.

The preparative HPLC chromatogram used for fractionation of SBE is shown in Fig. 1A. We then assessed the potential cytotoxicity of the different SBE fractions prepared using concentrations up to 50 μg/ml and found that the dose range tested did not reduce the OA chondrocytes viability over 24 h, as evaluated by the MTT assays (Fig. 1B).

The multi-factorial etiology of OA includes 3 major components: oxidative stress due to overproduction of ROS, inflammation as a result of increased expression of inflammatory mediators such as IL-6, COX-2 and matrix degradation due to activation of matrix degrading enzyme. Therefore, to examine the potential chondroprotective properties of SBE fractions, we investigated the effect of all four fractions (F1-F4) on the suppression of ROS production and expression of IL-6 and MMP-13 following stimulation with IL-1β in OA chondrocytes. To determine the effect of SBE fractions on IL-1β induced oxidative stress, cellular redox status was determined by measuring the levels of ROS in OA chondrocytes. Changes in the fluorescence of oxidation-sensitive dye DHR123 in the labeled cells represent changes in intracellular ROS levels. Treatment of OA chondrocytes with IL-1β significantly increased the DHR fluorescence indicating the increased production of ROS, whereas pretreatment with SBE fractions (F1, F3, and F4) significantly decreased the IL-1β induced ROS production with maximum suppression being observed with F4 (Fig. 1C). Further as reported previously^21^, gene expression analyses showed that treatment of OA chondrocytes with IL-1β significantly increased the expression of IL-6 and MMP-13 mRNA compared to controls (Fig. 1D,E). Interestingly, OA chondrocytes treated with various concentrations of SBE fractions (1–50 μg/ml) showed that F3 and F4 significantly suppressed the expression of IL-6 and MMP-13 in a dose dependent manner, with F4 being most effective in suppressing the IL-1 β induced mRNA expression of IL-6 and MMP-13 in OA chondrocytes. Based on the ability to suppress the IL-1β induced oxidative stress and expression of inflammatory mediators, F4 was selected for further studies and for the elucidation of molecular mechanism of the observed chondroprotective effect.

### F4 contains Wogonin as a major component

**Figure 2:**
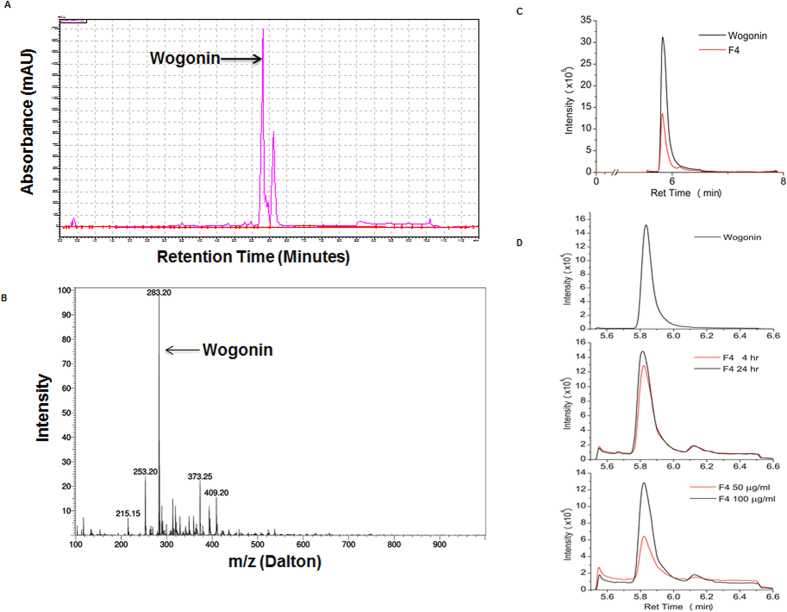
F4 contains Wogonin as a major component. (**A**) HPLC identification of Wogonin in F4. Fraction 4 (1 mg/ml) was subjected to HPLC on reversed-phase C 18 column as described in methods section. The major peak in HPLC chromatogram corresponds to Wogonin. (**B**) LC-MS/MS identification of Wogonin in F4. Fraction 4 (1 mg/ml) were directed to a mass spectrometer set up to run in Q1 scan using negative mode. Major peak at m/z 283.2 corresponds to Wogonin. (**C**) Quantification of Wogonin content in F4. F4 were directed to a mass spectrometer set up to run in negative multiple reaction (MRM-) mode detecting the transition pairs m/z 283.00 (precursor ion)/162.9 (product ion) for Wogonin. Quantification of Wogonin in F4 was calculated by four point calibration curves of Wogonin using MRM analysis. (**D**) Cellular uptake of F4 in OA chondrocytes. OA chondrocytes were treated with F4 (50, 100 μg/ml) for 4 or 24 h. Cell lysate were prepared and subjected to LC-MS/MS analysis as described in method section.

Since F4 exhibit most effective chondroprotective properties *in vitro* during the initial screening, we characterized the components present in F4 using HPLC and LC-MS/MS analyses. HPLC analysis identified the presence of Wogonin as a major component of F4 along with an additional small peak of unknown compound (Fig. 2A). Further, quantitative determination of the peaks using LC-MS/MS analysis confirmed Wogonin to be the predominant constituent of F4 with a molecular mass (m/z) of 283 (Fig. 2B). The other peaks in the MS chromatogram were at molecular mass of 253.2 and 373.25, which might be chryisn and neobaicalein or skullcapflavone II. Further, Wogonin content of F4 was quantified by four-point calibration curves of Wogonin using MRM analysis. Linear calibration curve with good correlation coefficients (R^2^ = 0.996) was obtained in the range of 10^−1^–10^−4^ mg/ml and Wogonin content in F4 was calculated to be 40% (Fig. 2C). Further, to investigate the intracellular concentration of F4 in treated chondrocytes, cellular uptake studies were performed using calibration curve of Wogonin by MRM analysis of lysates prepared from OA chondrocytes treated with F4 (50, 100 μg/ml) for 4 h or 24 h. It was observed that cellular uptake of F4 was increased in a time and dose dependent manner and intracellular concentration of Wogonin was calculated as 2.4 μg/ml when OA chondrocytes were treated with 100 μg/ml of F4 for 24 h (Fig. 2D). Altogether these results suggest Wogonin as the major constituent (40%) of F4 and membrane permeability of chondrocytes for the cellular uptake of F4 increases in a time and dose dependent manner.

### F4 inhibited the IL-1β induced oxidative and nitrosative stress in OA chondrocytes

**Figure 3:**
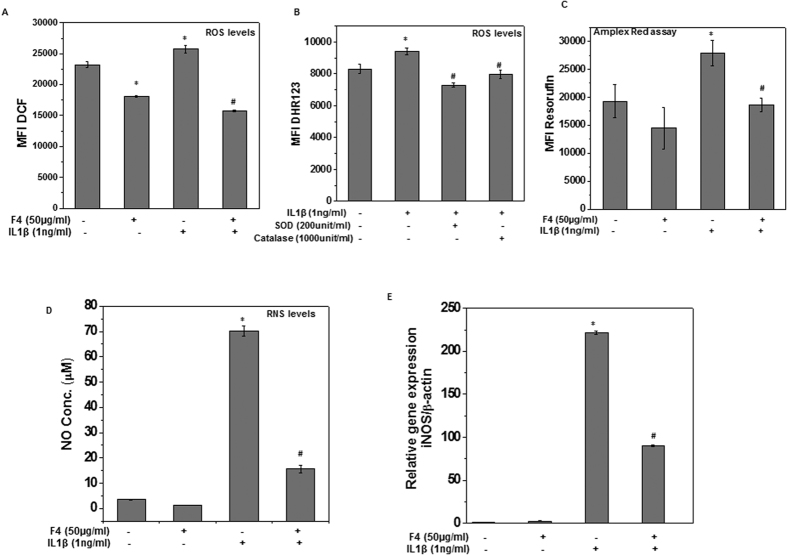
F4 inhibited the IL-1 β induced oxidative and nitrosative stress in OA chondrocytes. (**A**) F4 inhibited the IL-1β induced ROS production in OA chondrocytes. Human OA chondrocytes were treated with F4 (50 μg/ml) for 2 h, then stained with H2DCF-DA (20 μM) for 0.5 h at 37 °C, and stimulated with IL-1β (1 ng/ml) for 5 minutes at 37 °C. Fluorescence emission was measured at 525 nm. Bar graph shows relative fluorescence units indicating ROS levels. Data points represent mean ± SD from four replicates. *p ≤ 0.01, as compared to control, # ≤ 0.01, as compared to IL-1β.(**B**) IL-1β induced production of H2O2 and O2- in OA chondrocytes. OA chondrocytes were treated with catalase (1000 unit/ml) or SOD F4 (200 unit/ml) for 1 h, stained with DHR-123 (5 μM) for 0.5 h at 37 °C and then stimulated with IL-1β (1 ng/ml) for 5 minutes at 37 °C. Fluorescence emission was measured at 535 nm. Bar graph shows relative fluorescence units indicating ROS levels. (**C**) F4 inhibited the IL-1β induced production of H2O2 in OA chondrocytes. Human OA chondrocytes were treated with F4 (50 μg/ml) for 2 h, and the stimulated with IL-1β (1 ng/ml) for 5 minutes at 37 °C. H2O2 generation was estimated by Amplex red assay as described in method section. (**D–E**) F4 inhibited the IL-1β induced production of NO and expression of iNOS in OA chondrocytes. Primary human OA chondrocytes were pre-treated with F4 (50 μg/ml) for 2 h followed by treatment with IL-1β (1 ng/ml) for 16 h. At the end of treatment culture supernatant were collected and chondrocytes were harvested for RNA isolation. (**D**) NO was estimated in supernatant using Griess reagent as described in methods. (**E**) Expression of iNOS was measured by quantitative PCR using the TaqMan assay system (Life Technologies). β-actin was used as endogenous expression control. Bar graph represents mean ± SD from three subjects. *p ≤ 0.01, as compared to control, # ≤ 0.01, as compared to IL-1β.

**Figure 4:**
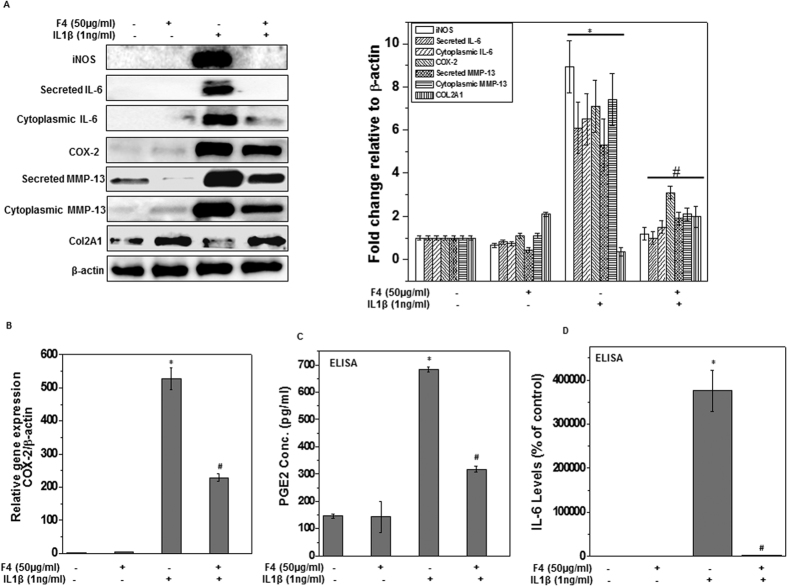
F4 inhibited IL-1 β the induced inflammatory mediators in OA chondrocytes. Primary human OA chondrocytes were pre-treated with F4 (50 μg/ml) for 2 h followed by treatment with IL-1β (1 ng/ml) for 16 h. At the end of treatment culture supernatant were collected and chondrocytes were harvested. Cell lysate were prepared using RIPA buffer for immunoblot analysis or RNA were isolated for real time PCR analysis. (**A**) Protein expression was investigated by immunoblotting using antibodies against indicated protein. β-actin was used as a control for equal loading. Specific signal intensities were quantified by ImageJ software. (**B**) Expression of COX-2 was measured by quantitative PCR using the TaqMan assay system. β-actin was used as endogenous expression control. (**C,D**) Secreted levels of IL-6 (**C**) and PGE2 production (**D**) were measured in the culture supernatant by ELISA. Bar graph represents mean ± SD from two subjects. *p ≤ 0.01, as compared to control, # ≤ 0.01, as compared to IL-1β.

Recent studies showed that oxidative and nitrosative stress due to excess generation of ROS and RNS (such as NO) activate the intracellular signaling processes that lead to chondrocytes apoptosis and ECM degradation and thus play a major role in OA progression^9^. Therefore, we determined whether F4 suppress IL-1β-induced oxidative and nitrosative stress in OA chondrocytes. The level of oxidative stress was determined by measuring the ROS level using oxidation-sensitive dye H_2_DCF-DA and it was found that pretreatment of chondrocytes with F4 significantly suppressed the basal as well as IL-1β induced ROS levels (Fig. 3A). To identify the nature of the ROS induced by IL-1β in OA chondrocytes, catalase or superoxide dismutase (SOD) were used prior to addition of IL-1β and it was observed that IL-1β mediated increase in DHR fluorescence was significantly suppressed in the presence of both catalase and SOD suggesting the involvement of H_2_O_2_ and O_2_^−^ (Fig. 3B). We specifically measured the H_2_O_2_ levels in treated chondrocytes using Amplex red assay and the results showed that pretreatment with F4 completely inhibited the IL-1β induced generation of H_2_O_2_ in OA chondrocytes (Fig. 3C). Further, to measure the effect of F4 on IL-1β mediated nitrosative stress, we examined the production of NO in the culture supernatants of treated chondrocytes using Griess reagent. In accordance with earlier published report^22^, our results showed that treatment of OA chondrocytes with IL-1β significantly increased the production of NO. Interestingly, OA chondrocytes treated with F4 prior to stimulation with IL-1β completely blocked the NO production suggesting the suppression of nitrosative stress by F4 (Fig. 3D). NO is produced by oxidation of L-arginine by inducible nitric oxide synthase (iNOS), an enzyme known to be upregulated by inflammatory cytokine IL-1β in OA chondrocytes^23^. Therefore, we examined whether F4 had any effect on IL-1β induced iNOS expression in OA chondrocytes. OA chondrocytes were treated with F4 (50 μg/ml) for 2 h and then stimulated with IL-1β (1 ng/ml) for 24 h and iNOS gene and protein expression levels were determined by TaqMan assays and immunoblot analysis, respectively. As shown previously^22^, in IL-1β-treated OA chondrocytes gene expression of iNOS mRNAs was several hundred fold higher than in control OA chondrocytes, whereas F4 pretreatment significantly suppressed the expression of iNOS mRNA (Fig. 3E).This suppression of gene expression was also reflected in completely suppressed protein levels of iNOS in OA chondrocytes despite the presence of IL-1β (Fig. 4A).These results indicated that F4 could effectively repress IL-1β-induced oxidative and nitrosative stress by suppressing the expression of iNOS and production of ROS and RNS in OA chondrocytes.

### F4 inhibited the IL-1β induced IL-6 and COX-2 expression in OA chondrocytes

Inflammation due to release of various inflammatory mediators such as IL-6 and COX-2 is recognized as important component of OA pathogenesis^24^. We determined whether F4 had any effect on IL-1β induced expression of IL-6 and COX-2. OA chondrocytes were pretreated with F4 for 2 h and then stimulated with IL-1β for 24 h and levels of gene and protein expression were determined by TaqMan assays and immunoblot analysis, respectively. Our results showed that F4 significantly suppressed IL-1β induced mRNA expression of IL-6 (Fig. 1D) and COX-2 (Fig. 4B) in OA chondrocytes. Further, Western blot analyses demonstrate that protein levels of COX-2 and IL-6 (both secreted and cytoplasmic) were also significantly suppressed by pre-treatment of human OA chondrocytes with F4 (Fig. 4A). Additionally, we also measured the secreted levels of IL-6 using sandwich ELISA and it was found that F4 completely blocked the secretion of IL-6 in culture supernatant of IL-1 β stimulated OA chondrocytes (Fig. 4C). COX-2 exerts its inflammatory effect through production of PGE_2_, which is a crucial molecule in a variety of physiological and pathological conditions including cartilage degradation^25^. Therefore, we examined the effect of F4 on IL-1β-induced PGE_2_ production and our results showed that production of PGE_2_, which increased following treatment with IL-1β, was also significantly suppressed by pretreatment of OA chondrocytes with F4 (Fig. 4D). Taken together, these results suggest that F4 may be an effective agent for blocking the IL-1β-induced inflammation by suppressing the levels of inflammatory mediators such as IL-6, COX-2 and PGE_2_.

### F4 inhibited the IL-1β induced matrix degradation in OA chondrocytes

**Figure 5:**
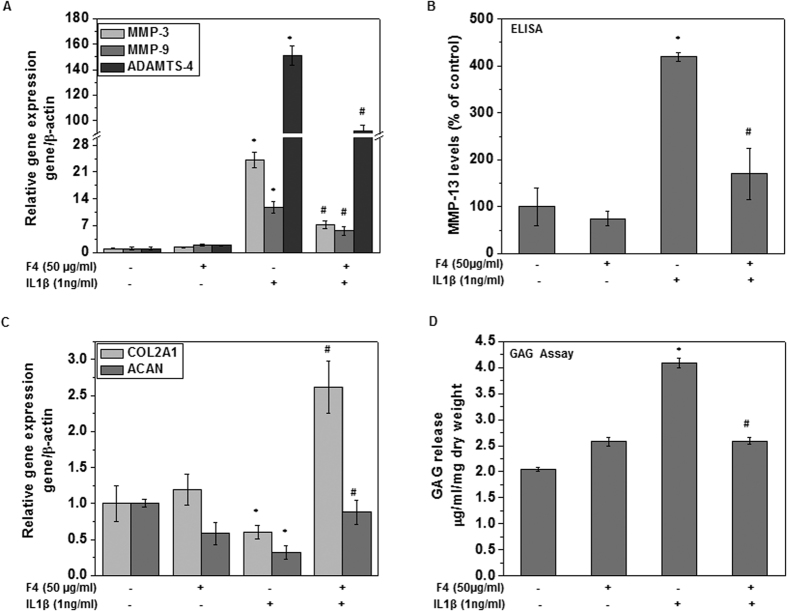
F4 inhibited the IL-1 β induced matrix degradation in OA chondrocytes. Primary human OA chondrocytes were pre-treated with F4 (50 μg/ml) for 2 h followed by treatment with IL-1β (1 ng/ml) for 16 h. At the end of treatment culture supernatant were collected and chondrocytes were harvested. RNA was isolated for real time PCR analysis. (**A**) Expression of MMP-3, MMP-9, and ADAMTS4 was measured by quantitative PCR using the TaqMan assay system. β-actin was used as endogenous expression control. (**B**) Secreted levels of MMP-13 were measured in the culture supernatant by ELISA. (**C**) Expression of COL2A1, and ACAN was measured by quantitative PCR using the TaqMan assay system. β-actin was used as endogenous expression control. (**D**). F4 inhibited the IL-1 β induced release of s-GAG in OA explants. Human OA cartilage pieces were incubated with F4 (50 μg/ml) for 2 h followed by treatment with IL-1β (25 ng/ml) for 72 h. The s-GAG release from cartilage explants in culture supernatants was quantified by using DMMB colorometric assay as described in methods section. Bar graph represents mean ± SD from two subjects. *p ≤ 0.01, as compared to control, # ≤ 0.01, as compared to IL-1β.

Matrix degradation due to activation of matrix degrading enzyme such as aggrecanases and metalloproteinases (MMPs) is characteristic feature of OA pathogenesis^26^. MMP-13, MMP-3, MMP-9 and ADAMTS-4 are the primary collagenases produced by chondrocytes in response to IL-1β that contribute to OA pathogenesis. Therefore, we investigated the effect of F4 on the IL-1β-induced mRNA expression of these MMPs and ADAMTS-4 in human OA chondrocytes. As shown in Figs 5A and 1E, IL-1β-stimulation of human OA chondrocytes resulted in significant up-regulation of ADAMTS-4, MMP-3, MMP-9 and MMP-13 mRNA expression whereas treatment with the F4 resulted in significant suppression of mRNA expression of all the these enzymes in OA chondrocytes (Figs 5A and 1E). To determine whether this inhibition of gene expression also influenced the MMP-13 protein production, culture supernatants were assayed for MMP-13 protein using a sandwich ELISA. A significant increase in secreted MMP-13 in the culture supernatant was found when OA chondrocytes were stimulated with IL-1β (Fig. 5B). However, pretreatment of chondrocytes with F4 significantly inhibited the MMP-13 production in IL-1β-stimulated OA chondrocytes (Fig. 5B). Additionally, Western blot analysis further demonstrates that protein levels of MMP-13 (both secreted and cytoplasmic) were also significantly suppressed by pre-treatment of human OA chondrocytes with F4 (Fig. 4A).

Since ADAMTS-4 and MMP-13 cause ECM degradation by cleavage of aggrecan and collagen respectively, we explored if the F4 affected the ACAN and COL2A1 gene expression in chondrocytes stimulated with IL-1β. IL-1β-stimulation resulted in significant down regulation of ACAN and COL2A1 expression in OA chondrocytes (Fig. 5C). However, pretreatment with F4 significantly blocked the IL-1β-mediated down regulation of ACAN and COL2A1 compared to IL-1β alone stimulated OA chondrocytes (Fig. 5C).

Since activation of matrix degrading enzymes results in aggrecan degradation leading to cartilage matrix destruction in the form of release of sulfated glycosaminoglycans (s-GAGs), hence, the loss of s-GAGs is considered as a crucial parameter to determine the progression of cartilage destruction. Therefore, to evaluate the effect of F4 on IL-1β-induced release of cartilage matrix molecules, the level of s-GAGs in the cartilage explants culture media was examined. Results showed that IL-1β-induced human cartilage matrix degradation was significantly increased in the culture medium of cartilage explants treated with IL-1β, compared to un-stimulated control cartilage explants (Fig. 5D). However, IL-1β-induced release of s-GAG was significantly inhibited by pretreatment of human cartilage explants with F4 (Fig. 5D).Taken together these results provide support that F4 could be a potent cartilage chondroprotective agent for effective suppression of IL-1β-mediated expression of matrix degrading enzymes and release of s-GAGs from human cartilage.

### F4 did not inhibit the IL-1β induced activation of MAPKs in OA chondrocytes

**Figure 6:**
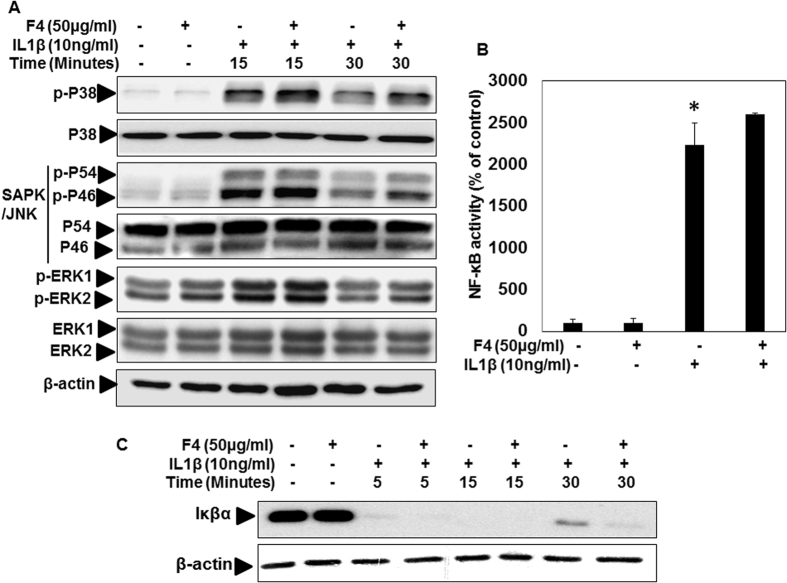
F4 did not inhibit the IL-1 β induced activation of MAPKs and NF-κB in OA chondrocytes. Primary human OA chondrocytes were pre-treated with F4 (50 μg/ml) for 2 h followed by treatment with IL-1β (10 ng/ml) for 15 or 30 minutes. Cell lysate were prepared from harvested chondrocytes using RIPA lysis buffer or nuclear extracts were prepared using nuclear extract kit (Active Motif). (**A**) Activation of MAPKs was investigated by immunoblotting using primary antibodies specific for phospho-ERK1/2, phospho-JNK, phospho-p38. Expression of total ERK1/2, total JNK, or total-p38 was used as control. Immunoblot results are representatives of two blots performed on samples obtained from two individuals. (**B**) Binding activity of NF-κB from nuclear extracts of the cells treated as above to its consensus sequence was quantitated by a specific ELISA assay (Active Motif). (**C**) Degradation of Iκβα was investigated by immunoblotting in cell lysate prepared as above. β-actin was used as a control for equal loading. Bar graph represents mean ± SD from two subjects. *p ≤ 0.01, as compared to control.

IL-1β-induced expression of MMPs has been shown to be mediated by activation of the family of MAPKs including ERK, p38 kinase, and JNK^27^,^28^,^29^,^30^. To evaluate the mechanisms responsible for the F4 mediated suppressive effects, we examined the effects of F4 on MAPK signaling cascade by measuring the phosphorylation levels of ERK, JNK and p38 in IL-1β stimulated OA chondrocytes. Results illustrated that stimulation of OA chondrocytes with IL-1β activates the phosphorylation of p38, JNK and ERK1/2 within 15 minutes of treatment followed by a slow decline at 30 minutes (Fig. 6A). Surprisingly, pretreatment of OA chondrocytes with F4 for 2 h prolonged the phosphorylation of p38, JNK and ERK1/2 stimulated by IL-1β (Fig. 6). The stimulatory effect with F4 for the phosphorylation of p38, JNK and ERK1/2 was found to be more prominent at 30 minutes of IL-1β stimulation (Fig. 6A). Taken together these results indicate that F4 did not inhibit the activation of MAPK signaling cascade indicating that the suppressive effect of F4 on MMPs expression was independent of IL-1β activated MAPK signaling pathways in OA chondrocytes.

### F4 inhibited the IL-1β induced activation of c-Fos/AP-1 but not of NF-κB

Since IL-1β induced inflammatory mediators such as IL-6, COX-2 and iNOS are transcriptionally regulated by AP-1 and NF-κB transcription factors^5^,^21^,^31^, therefore, we examined the possible inhibitory effects of F4 on DNA-binding activity of AP-1 and NF-κB in IL-1β stimulated OA chondrocytes. We first examined the effect of F4 on the activation of NF-κB in IL-1β-stimulated OA chondrocytes. As indicated by DNA binding activity, NF-κB was strongly activated by IL-1β, but F4 did not inhibit the DNA-binding activity of p65/NF-κB (Fig. 6B). Besides modifying p65, stimulation with IL-1β causes the proteasomal degradation of IκBα resulting in the release and translocation of NF-κB protein to the nucleus^25^. Therefore, we also evaluated the effect of F4 on degradation of IκBα and it was found that stimulation with IL-1β alone caused degradation of IκBα within 5 minutes of treatment, which was not prevented by F4 indicating that F4 did not block the activation of NF-κB in IL-1β-stimulated OA chondrocytes (Fig. 6C).

**Figure 7:**
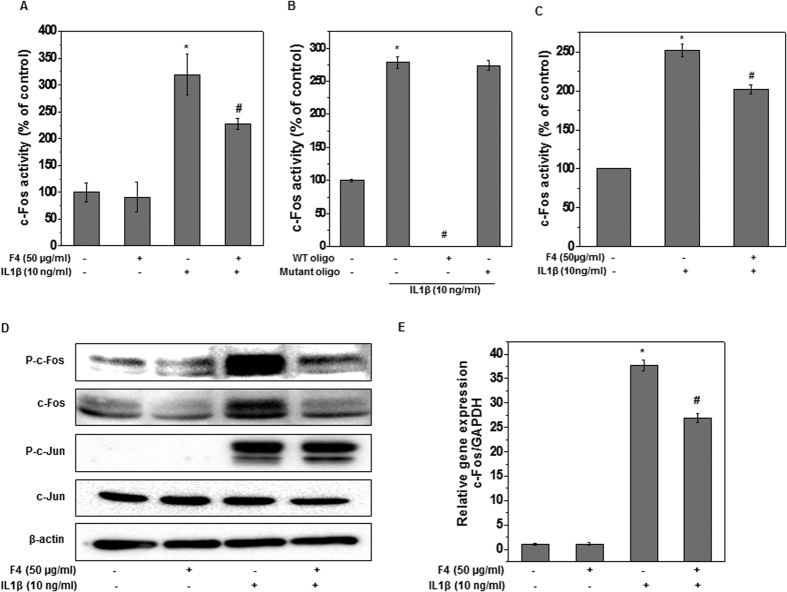
F4 inhibited the IL-1β induced activation of c-Fos/AP-1. Primary human OA chondrocytes were pre-treated with F4 (50 μg/ml) for 2 h followed by treatment with IL-1β (10 ng/ml) for 30 minutes. Cell lysate were prepared from harvested chondrocytes using RIPA lysis buffer or nuclear extracts were prepared using nuclear extract kit (Active Motif). (**A,B**) Binding activity of c-Fos/AP-1 from nuclear extracts of the cells treated as above to its consensus sequence was quantitated by a specific ELISA assay (Active Motif). (**C**) F4 (50 μg/ml) was added to nuclear extracts of the IL-1β treated cells for 0.5 h and binding activity of c-Fos/AP-1 was quantitated by a specific ELISA assay (Active Motif). (**D**) Protein expression was investigated by immunoblotting using antibodies against indicated protein. β-actin was used as a control for equal loading. (**E**) Expression of c-Fos was measured by quantitative PCR using the TaqMan assay system. GAPDH was used as endogenous expression control. Immunoblot results are representatives of two blots performed on samples obtained from two individuals. Bar graph represents mean ± SD from two subjects. *p ≤ 0.05, as compared to control, # ≤ 0.05, as compared to IL-1β.

We next examined the effect of F4 on the activation of AP-1 and it was found that in corroboration with an earlier report^32^, our result showed significant induction of c-Fos/AP-1 DNA-binding activity after IL-1β stimulation in OA chondrocytes (Fig. 7A). Interestingly, pretreatment of OA chondrocytes with F4 significantly suppressed the IL-1β induced c-Fos activity (Fig. 7A). The c-Fos/AP-1 DNA binding complex was specific because it could be competed by wild-type but not by mutant AP-1 oligonucleotides (Fig. 7B). Since some of AP-1 inhibitors inhibit AP-1 activation by directly blocking the binding of AP-1 to the DNA, therefore, to examine whether F4 directly suppressed AP-1 binding to its cognate oligonucleotides, we incubated nuclear extracts from IL-1β stimulated OA chondrocytes with F4 and then performed c-Fos activity assay. The result showed that F4 directly interfered with the DNA binding of c-Fos/AP-1 indicating that F4 might inhibit the binding of AP-1 to the DNA through modification of c-Fos proteins (Fig. 7C).

Since, AP-1 is mainly formed by homo or hetero dimerization of c-Fos and c-Jun, we therefore, wondered whether F4 may transcriptionally or post-transcriptionally regulate the levels of c-Fos or c-Jun. OA chondrocytes were treated with F4 in the presence or absence of IL-1β and the total cell lysates were used to determine levels of c-Fos or c-Jun by western blot. The results indicated that F4 potently reduced theIL-1β induced phosphorylation and total c-Fos levels in OA chondrocytes (Fig. 7D). However, under similar condition, the protein level of c-Jun was not affected by treatment with F4 (Fig. 7D). Further, to evaluate the effect at transcriptional level, we examined the effects of F4 on mRNA expression of c-Fos in IL-1β stimulated OA chondrocytes using TaqMan assay. The results showed that F4 effectively suppressed the IL-1β induced c-Fos mRNA expression in OA chondrocytes (Fig. 7E). The results, therefore, suggest that F4 regulates c-Fos expression, through transcriptional and post-transcriptional mechanisms. Altogether, these results suggest that F4 exhibits chondroprotective effects through suppression of c-Fos and DNA binding activity of AP-1 in IL-1β stimulated OA chondrocytes.

### Purified Wogonin exerts similar chondroprotective effects in OA chondrocytes

**Figure 8:**
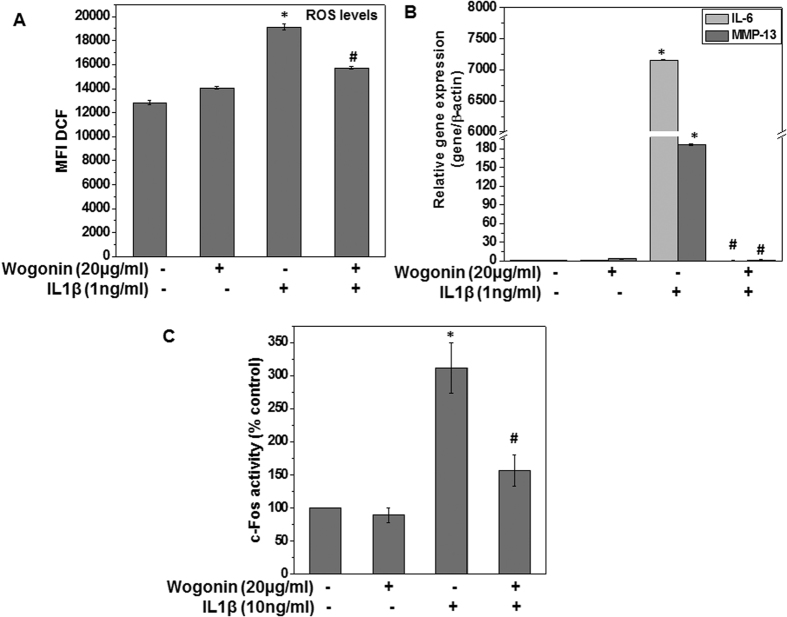
Purified Wogonin exerts chondroprotective effects in OA chondrocytes. (**A**) Human OA chondrocytes were treated with purified Wogonin (20 μg/ml) for 2 h, then stained with H_2_DCF-DA (20 μM) for 0.5 h at 37 °C and stimulated with IL-1β (1 ng/ml) for 5 minutes at 37 °C. Fluorescence emission was measured at 525 nm. Bar graph shows relative fluorescence units indicating ROS levels. Data points represent mean ± SD from four replicates. *p ≤ 0.01, as compared to control, # ≤ 0.01, as compared to IL-1β. **(B)** Primary human OA chondrocytes were pre-treated with purified Wogonin (20 μg/ml) for 2 h followed by treatment with IL-1β (1 ng/ml) for 16 h. At the end of treatment culture chondrocytes were harvested and RNA was isolated for real time PCR analysis. Expression of IL-6 and MMP-13 was measured by quantitative PCR using the TaqMan assay system. β-actin was used as endogenous expression control. **(C)** Primary human OA chondrocytes were pre-treated with purified Wogonin (20 μg/ml) for 2 h followed by treatment with IL-1β (10 ng/ml) for 30 minutes and nuclear extracts were prepared using nuclear extract kit (Active Motif). Binding activity of c-Fos/AP-1 to its consensus sequence was quantitated by a specific ELISA assay (Active Motif). Bar graph represents mean ± SD from two subjects. *p ≤ 0.01, as compared to control, # ≤ 0.01, as compared to IL-1β.

Since Wogonin was major constituent of F4, we wondered whether observed effects of F4 may be attributed to Wogonin. Therefore, purified Wogonin was used to determine its suppressive effects in IL-1β treated OA chondrocytes. For this study, 20 μg/ml of Wogonin, the concentration found in F4, was used and the effect of Wogonin on the IL-1β-induced production of ROS, expression of IL-6 and MMP-13 and activation of AP-1/c-Fos in human OA chondrocytes were analyzed. Results showed that treatment with Wogonin significantly inhibited the IL-1β-induced production of ROS and expression of IL-6 and MMP-13 mRNA in OA chondrocytes (Fig. 8A,B). Further, purified Wogonin suppressed the IL-1β-induced activation of c-Fos in OA chondrocytes (Fig. 8C). Altogether, these results suggest that Wogonin exerts chondroprotective effects by suppressing the production of ROS and expression of inflammatory mediators through the inhibition of c-Fos in IL-1β stimulated OA chondrocytes. Taken together these results strongly imply that the observed cartilage chondroprotective effects of F4 could be attributed to the effect of its predominant component Wogonin.

## Discussion

Osteoarthritis is a heterogeneous complex joint disease with gradual degradation of cartilage matrix due to the cleavage of ECM components including collagen type II, and proteoglycans. Pro-inflammatory cytokines IL-1β and TNF-α are believed to be the key proteins responsible for the initiation and progression of cartilage degeneration^4^. Current treatment options for OA include NSAIDs and acetaminophen, however long term uses of these drugs have severe side effects such as gastrointestinal ulcers and cardiovascular adverse events^5^. Due to these limitations, use of nutraceuticals for the management of OA is on the rise^33^. Nutraceuticals derived from herbs, spices and medicinal plants rich in polyphenols are the most widely studied natural health products with relevance to OA^11^. Some of these compounds such as resveratrol, curcumin and EGCG have been shown to exert positive effects for OA^33^. Extracts from the roots of *S. baicalensis*, a traditional Chinese medicine is used for the treatment of inflammatory and cardiovascular disorders^12^. Recent reports showed that *S. baicalensis* extract in combination with *A. catechu* alleviated joint discomfort and reduced the stiffness and the production of pro-inflammatory molecules in OA patients^16^,^17^. However, detailed mechanism and active constituents of the preparations administered were not identified in these studies. In the present study, chondroprotective potential and the constituents of an active fraction of root extract of *S. baicalensis* were identified and the fraction was evaluated with respect to its ability to suppress the spectrum of key molecules released during OA pathogenesis. Our results provide evidence of cartilage protection via suppression of molecular events of inflammation, oxidative stress and matrix degradation by active fraction F4 in IL-1β-stimulated OA chondrocytes and cartilage explants.

Inflammation has now emerged as an important mediator of OA pathogenesis as high levels of inflammatory cytokines such as IL-1β, TNF-α, and IL-6 were found in the OA joint^5^,^6^. IL-1β is a key cytokine involved in OA pathogenesis and considered as the internal regulator of cartilage matrix degradation because of its ability to stimulate the expression of other inflammatory mediators such as MMPs, IL-6, COX-2, PGE_2_, iNOS and NO^34^,^35^,^36^. Water extract of *S. baicalensis* has previously been shown to inhibit the expression of IL-6, COX-2 and iNOS and production of PGE_2_ and NO in culture supernatant of LPS stimulated Raw 264.7 cells^12^,^34^. In the present study, we identified Wogonin to be the dominant constituent of F4 and used this Wogonin rich fraction (F4) of an ethanolic extract of *S. baicalensis* to suppress the IL-1β-stimulated production of inflammatory mediators in OA chondrocytes (Figs 3D,E and 4A–D). The inhibitory effect of F4 on IL-1β induced NO production is interesting as inhibition of NO has been associated with the reduced pain, inflammation, proteoglycan loss in animal models of arthritis and in human OA patients^35^,^36^. Our results showing the inhibitory effects of F4 on IL-1β-induced ROS generation (Fig. 3A,C) confirmed the previous findings of antioxidant potential of *S. baicalensis* extract^37^, and thus provide support to the use of antioxidant and nutraceuticals in the management of OA.

MMP-13 produced by chondrocytes in response to IL-1β is the primary collagenase involved in OA pathogenesis. MMP-3 and MMP-9 are other members of this family known to be involved in the pathogenesis of OA. The use of specific inhibitors of MMPs as novel treatment strategy for OA has been highlighted in recent studies^38^. Therefore, we studied the effects of F4 on the production of MMPs in human OA chondrocytes and the results showed that F4 potently down regulates the IL-1β induced expression of MMP-13, MMP-3 and MMP-9 and the secreted levels of MMP-13 (Figs 4A and 5A,B). Interestingly, our data showed that treatment of OA chondrocytes with F4 in the presence of IL-1β up-regulated the expression of cartilage anabolic factors such as COL2A1 and ACAN (Fig. 5C) which are known to be suppressed by IL-1β^22^. Our results also showed that F4 also inhibited the IL-1β-induced s-GAG release from cartilage explants *in vitro* (Fig. 5D). Aggrecan is degraded predominantly by aggrecanases and MMPs, thus observed inhibition of s-GAG release by F4 may possibly be due to inhibition of MMPs and ADAMTS4. Moreover, induction of COL2A1 and ACAN expression by F4 suggests that this Wogonin rich fraction has potential to reverse the metabolic axis of chondrocytes from catabolic towards anabolic direction and thus inhibit the disease progression.

The data presented in the current study revealed that inflammatory and oxidative molecules such as IL-6, COX-2, iNOS, MMP-13, NO and ROS are major targets of F4 in OA chondrocytes. Earlier studies from our and other laboratories have established the transcriptional regulation of these inflammatory mediators under the control of transcription factors NF-κB, and AP-1^21^,^28^. IL-1β exerts its effects on the expression of a number of genes by mainly activating NF-κB and AP-1 transcription factors leading to inflammation and joint destruction^39^. To dissect the molecular signature of F4 mediated chondroprotective/anti-inflammatory effects, we examined the modulation of these transcription factors by F4 in OA chondrocytes stimulated with IL-1β. F4 had no effect on NF-κB activation and DNA binding activity in IL-1β stimulated OA chondrocytes (Fig. 6B,C). The inhibitory effects of F4 on the expression of inflammatory mediators were chiefly mediated by the suppression of expression, phosphorylation, activation and DNA binding activity of c-Fos in IL-1β-stimulated OA chondrocytes. Further, c-Fos appears to be specific target of F4 for its chondroprotective effect, as c-Jun, the other major component of AP-1 was not affected by F4 in IL-1β-stimulated OA chondrocytes. These are novel differential effect of F4 or Wogonin on AP-1 components which has not been reported previously. AP-1 is a major transcription factor that directly controls the expression of several inflammatory cytokines, chemokines, and matrix metalloproteinases (MMPs) by binding directly to AP-1 motifs in the promoter region of these genes^40^. Our results showed that F4 had strong potential of binding the c-Fos promoter, therefore inhibiting DNA binding to cognate oligonucleotides and thus activation of c-Fos/AP-1 in OA chondrocytes (Fig. 7C). Thus, F4 might be regulating the activity of c-Fos at transcription and post transcription levels by directly interfering with the DNA binding of c-Fos/AP-1. Since the suppressive effects of F4 were mimicked by pure Wogonin in some of the studies suggests that the active component of F4 was Wogonin.

In conclusion, here we demonstrates that a Wogonin rich fraction (F4) isolated from root extract of *S. baicalensis* inhibits the IL-1β-stimulated levels of oxidative stress mediators (ROS, NO), expression and production of inflammatory mediators (IL-6, COX-2, PGE2, iNOS and NO), major proteases (MMP-3, MMP-9, MMP13, ADAMTS4) and s-GAG release associated with cartilage degradation. We also showed cartilage protective ability of F4 by up-regulating cartilage anabolic factors like COL2A1 and ACAN in OA chondrocytes. We are reporting for the first time that purified Wogonin and Wogonin rich fraction (F4) inhibit the phosphorylation and activation of c-Fos/AP1 in IL-1β-stimulated human OA chondrocytes, thereby blocking the activation of downstream signaling events that lead to the production inflammatory mediators. Thus, our results suggest that constituents of SBE or components derived from it may possess chondroprotective properties and may be of value in inhibiting the induction and/or pathogenesis of OA. Thus, with this unique profile, Wogonin rich active fraction of SBE may prove to be a potentially attractive product in the management of OA.

## Material and Methods

### Reagents

Media and other reagents for cell culture were purchased from HyClone Laboratories (Logan, UT, USA) or from Life Technologies (Carlsbad, CA, USA). Pronase and Collagenase were from Roche Diagnostics (Indianapolis, IN, USA). Recombinant human IL-1β was from R&D Systems (St Paul, MN, USA). Antibodies specific for p-ERK1/2, ERK1/2, p-JNK, JNK, p-P38, P38, p-c-Fos, c-Fos, p-c-Jun, c-Jun, and Iκβα were purchased from Cell Signaling Technology (Beverly, MA). Antibodies specific for β-Actin, MMP-13, IL-6, iNOS, and COL2A1 were from Santa Cruz Biotechnology (Santa Cruz, CA, USA). Anti-COX-2 antibody was from R&D Systems. Baicalein, Baicalin, Wogonin, Scutellarein and Apigenin were purchased from Extrasynthese (GenayCedex, France).

### Preparation of *Scutellaria baicalensis* extract (SBE)

The dried roots of *S. baicalensis* were purchased from the Mayway Hebei, Mayway’s joint-venture herb processing facility (Anguo, China). The dried roots were ground to powder and the extract (SBE) was prepared as described previously^13^. In brief, the dried powder (10 g) of *S. baicalensis* root was extracted with 70% ethanol in a Soxhlet extractor for 20 h. Removal of the solvent was done under reduced pressure in a rotary evaporator (Rotavapor; BUCHI Labortechnik AG, Flawil, Switzerland) and was dried using the Labconco FreeZone 2.5 freeze dryer (Labconco, Kansas City, MO). We obtained 3.61 g of the extract powder (36.1% yield), which was stored desiccated at 4 °C.

### Activity guided fractionation of SBE

The extract was fractionated on Prominence preparative HPLC system (Shimadzu Corporation, Kyoto, Japan) and chromatographic separation was performed at 40 °C using a Luna C18 preparative column (5 μm particle size, 150 cm L × 21.20 mm I.D.; Phenomenex, Torrance, CA). HPLC separations for time-based and peak-based fractionation were carried out with H_2_O (A) and methanol +0.1% Formic acid (B) and, at a flow rate of 10 ml/min. The gradient profile was as follows: 5% to 50% methanol in 5 minutes, followed by 95% methanol for 30 minutes. Fraction 1 (F1) was collected between 7.5 to 15 minutes of elution time, whereas fraction 2 (F2), fraction 3 (F3) and fraction 4 (F4) were collected from 15.2–17 minutes, 17.2–20 minutes and 20.5–23.5 minutes of elution respectively (Fig. 1A). After evaporation of the solvent, all fractions were freeze-dried under reduced pressure and dry powder was dissolved in methanol at a concentration of 1 mg/ml and was used for analysis using HPLC and LC-MS/MS.

### Characterization and quantification of bioactive compound in F4 by HPLC and LC-MS/MS analysis

Identification of F4 constituents was carried out using UHPLC on reversed-phase C 18 column. Solvent (A) was water and solvent (B) was acetonitrile (with 0.1% Formic acid) and a flow rate of 1 ml/min was maintained (initial 15%B, then 0–4 min 30% B; 4–8 min 30% −50% B; 8–10 min 95% B; 10–12 min 95% to 15%B). Baicalein, Baicalin, Wogonin, Scutellarein and Apigenin standard were diluted in methanol at a concentration of 1 mg/ml and were found to elute at 4.39, 2.34, 5.83, 2.56 and 4.03 min respectively in HPLC analysis using the described parameters.

Further characterization of eluted compounds in F4 was performed by LC-MS/MS using a UHPLC system connected to a triple quadrupole mass spectrometer (LC-MS 8040; Shimadzu, Kyoto, Japan) equipped with an electrospray ionization source. Chromatographic separation was performed using a Kinetex EVO C-18 analytical column (1.7 μm particle size, 100 mm L 2.1 mm I.D.; Phenomenex, Torrance, CA). Mobile phase A was 100% water and mobile phase B was 100% acetonitrile containing formic acid (0.1%, v/v). The component in F4 was identified and quantitated using the ESI ion source in multiple reaction monitoring and negative ion mode (MRM-) by monitoring transition pairs m/z 283.00 (precursor ion)/162.9 (product ion). The following instrument settings were used for MRM analysis: heat block temperature, 400 °C; DL temperature, 250 °C; nebulizing gas (N_2_), 3 L/min; drying gas (N_2_), 15 L/min; collision energy, 35.0; dwell time, 100 msec. A calibration curve was prepared using pure Wogonin dissolved in methanol at the concentrations of 0.001, 0.005, 0.01, 0.1 and 1 mg/ml.

The mass spectrometric analysis for Wogonin in cell lysate treated with F4 was performed using LC-MS/MS MRM- analysis. The cell lysate were prepared as described earlier^41^.

### Preparation of primary human chondrocytes and treatment with SBE fractions

Prior to the initiation of the studies the study protocol was reviewed and approved by the Institutional Review Board (IRB) of Northeast Ohio Medical University, Rootstown, Ohio as a “non-human subject study under 45 CFR” and that no informed consent was needed. All the methods used in this study were carried out in accordance with the approved guidelines and all experimental protocols were approved by the IRB of Northeast Ohio Medical University, Rootstown, Ohio. Discarded and de-identified knee or hip joint cartilage samples were collected through the NIH supported National Disease Research Interchange (NDRI) per the IRB approved protocol. The unaffected areas of the cartilage was used as source to isolate OA chondrocytes by enzymatic digestion as previously described^21^,^25^. Primary human OA chondrocytes (1 × 10^6^/well of 6-well plate) were seeded in Dulbecco’s modified Eagle’s medium DMEM)/Nutrient Mixture F-12 (DMEM/F-12) supplemented with 10% fetal calf serum (FCS) (Life Technologies), 100 units/ml penicillin, and 100 mg/ml streptomycin (Sigma) for 2–3 days after plating. At about 80% confluence, OA chondrocytes were serum starved overnight and were then treated with different concentration of SBE fractions dissolved in DMSO (Sigma) for 2 h followed by stimulation with IL-1β (1 ng/ml). Chondrocytes treated with 0.1% DMSO served as control.

### Cell viability assay

Chondrocytes viability was determined using MTT assay. Primary human OA chondrocytes were seeded in a 96-wells plate at a density of 20,000 cells per well in 200 μl complete medium. At confluence, chondrocytes were serum starved overnight and then treated with different concentrations of SBE fractions (1, 10, 50 μg/ml) for 24 h in serum free medium. Chondrocytes were further treated with 20 μl of 0.5% MTT (Sigma) for 4 h, and formazan crystal were solubilized by 150 μl of DMSO. Absorbance was recorded at 570 nm using the Synergy H1 multi-mode plate reader (Bio-Tek Instruments Inc., Winooski, VT, USA).

### Reactive oxygen species (ROS) measurement

Basal and induced ROS levels were measured by using cell-permeable fluorogenic probes DHR123 (Santa Cruz Biotechnology) and H_2_DCF-DA (Sigma) as described previously^42^. For the measurement of IL-1β induced ROS, chondrocytes were pretreated with SBE fractions (50 μg/ml) for 2 h and then labeled with DHR123 (5 μM) or H_2_DCF-DA (20 μM) for 0.5 h followed by stimulation with IL-1β (1 ng/ml) for 5 minutes. Cell suspension of chondrocyte was prepared and ROS levels were estimated by measuring the fluorescence emission Rhodamine123 or DCF at an excitation and emission wavelengths of 500 and 536 nm or 485 and 525 nm respectively using plate reader.

### Reactive nitrogen species (RNS) measurement

NO in the culture supernatant of treated chondrocytes was estimated using Greiss reagent as by described earlier^42^. Culture supernatant (50 μl) was incubated 50 μl of Greiss reagent (1%sulfanilamide, 0.1%N- (1-naphthyl)-ethylene-diamine hydrochloride (Sigma), 2.5%phosphoric acid in distilled water). The absorbance was measured at 550 nm using plate reader.

### Amplex red assay

For the measurement of IL-1β induced H_2_O_2_ generation, OA chondrocytes were pretreated with F4 (50 μg/ml) for 2 h followed by stimulation with IL-1β (1 ng/ml) for 30 minutes and assay was performed as per the manufacturer’s protocol (Thermo Fischer Scientific).

### Total RNA isolation and real time PCR

Chondrocytes were pretreated with F4 (50 μg/ml) for 2 h followed by treatment with IL-1β (1 ng/ml) for 24 h. Total RNA from isolated chondrocytes and cDNA was synthesized as described previously^25^. The mRNA expression of MMP-13, MMP-3, MMP-9, IL-6, iNOS, COX-2, ADAMTS4, COL2A1, ACAN was quantified using TaqMan Gene Expression Assays (Integrated DNA technology) as previously described^25^. Relative expression levels were calculated using the 2−ΔΔC_T_ method^43^.

### Western Immunoblotting

After treatments, OA chondrocytes were harvested, washed with cold PBS and lysed in ice-cold RIPA buffer as described previously^25^. Equivalent amounts of lysate protein (20 μg) were resolved by SDS-PAGE, transferred and blot were developed as described earlier^25^.

### Estimation of IL-6, MMP-13 and PGE_2_ in the supernatant using ELISA

After the treatment of chondrocytes, culture supernatants were collected and concentration of IL-6, MMP-13 and PGE_2_ was estimated using a commercially available ELISA (Boster Immunoleader, Pleasanton, CA or R&D system, St Paul, MN).

### Measurement of c-Fos/AP-1 or NF-κB DNA binding activity by ELISA

Activation of c-Fos/AP-1 or NF-κB and DNA-binding activity assay was performed using Trans-AM ELISA kit (Active Motif, Carlsbad, CA). Briefly, chondrocytes were treated as described above for the indicated times and nuclear extracts were prepared using the nuclear extract kit (Active Motif). A total 15 μg of the nuclear extract was used to determine the activity of c-Fos/AP-1 or NF-κB as per manufacturer’s instruction.

### GAG release assay

Cartilage explants were obtained from femoral condyles of OA cartilage samples. After weighing, the explants were transferred to a 24-well plate and cultured in basal medium for 24 h, serum starved overnight and then treated with F4 (50 μg/ml) for 2 h and then stimulated with IL-1β (25 ng/ml) for 72 h. The release of s-GAG was estimated in supernatant using DMMB (Sigma) assay as described earlier^44^.

### Statistical Analyses

The values are presented as Mean ± SD and the statistically significant difference between experimental groups and controls were analyzed using one-way-ANOVA followed by post hoc analyses using Tukey-test. Each experiment was repeated three times using three independent samples. *P* < 0.05 was considered statistically significant.

## Additional Information

**How to cite this article**: Khan, N. M. *et al*. A Wogonin-rich-fraction of *Scutellaria baicalensis* root extract exerts chondroprotective effects by suppressing IL-1β-induced activation of AP-1 in human OA chondrocytes. *Sci. Rep.* **7**, 43789; doi: 10.1038/srep43789 (2017).

**Publisher's note:** Springer Nature remains neutral with regard to jurisdictional claims in published maps and institutional affiliations.
